# Role of C17 fengycin B from *Bacillus subtilis* DB9011 in enterotoxigenic *Escherichia coli* inhibition

**DOI:** 10.1128/aem.00276-26

**Published:** 2026-06-04

**Authors:** Yuki Indo, Kosuke Shimizu, Ayane Sato, Fu Namai, Weichen Gong, Julio Villena, Chie Hikita, Keita Nishiyama, Haruki Kitazawa

**Affiliations:** 1 Laboratory of Animal Food Function, Graduate School of Agricultural Science, Tohoku University 74212 , Sendai, Japan; 2 SDS Biotech K.K., Tsukuba Research & Technology Center 73461 , Tsukuba, Japan; 3 Livestock Immunology Unit, International Education and Research Centre for Food and Agricultural Immunology (CFAI), Graduate School of Agricultural Science, Tohoku University 74212 , Sendai, Japan; 4 Division of Animal Immunology and Omics, CFAI, Graduate School of Agricultural Science, Tohoku University 267774 , Sendai, Japan; Universidad de los Andes 27991, Bogotá, Colombia

**Keywords:** *Bacillus subtilis *DB9011, fengycin B, lipopeptides, antimicrobial metabolites, enterotoxigenic *Escherichia coli*, swine production

## Abstract

Enterotoxigenic *Escherichia coli* (ETEC) is a leading cause of post-weaning diarrhea in piglets, and the rise of antimicrobial resistance underscores the urgent need for antibiotic alternatives in swine production. *Bacillus* species are promising candidates owing to their ability to produce antimicrobial secondary metabolites, particularly cyclic lipopeptides such as surfactins, iturins, and fengycins. Although *Bacillus* lipopeptides are known to exhibit antibacterial activity against *E. coli*, the specific compounds responsible for the inhibition of ETEC in probiotic-relevant *Bacillus* strains remain undefined. Here, we sought to identify extracellular metabolite(s) associated with the anti-ETEC activity of *Bacillus subtilis* DB9011. DB9011 culture supernatant strongly inhibited the growth of ETEC and other enteric pathogens, including *Salmonella* Typhimurium. Bioactivity-guided fractionation of the culture supernatant, followed by liquid chromatography–quadrupole time-of-flight mass spectrometry, identified C17 fengycin B as a major active metabolite enriched in inhibitory fractions, but absent from the non-inhibitory type strain NBRC13719ᵀ. A fengycin-containing fraction enriched in C17 fengycin B exhibited concentration-dependent antibacterial activity against ETEC and enhanced growth inhibition when combined with colistin, whereas incomplete inhibition at native supernatant-equivalent concentrations indicated that additional metabolites contribute to the overall antibacterial phenotype. While fengycins have been primarily recognized for antifungal activity and, more recently, for effects on gram-negative bacteria, our findings identify C17 fengycin B as a major contributor to the ETEC-inhibitory phenotype of *B. subtilis* DB9011. These findings provide mechanistic insights into how probiotic-relevant *Bacillus* strains inhibit enteric pathogens; nonetheless, further studies are required to evaluate *in vivo* relevance and functional performance under intestinal conditions.

**IMPORTANCE:**

Antimicrobial resistance poses a major threat to livestock production, where antibiotics remain widely used to control post-weaning diarrhea caused by enterotoxigenic *Escherichia coli* (ETEC). Supplementation with *Bacillus subtilis* DB9011 is known to reduce pathogenic *E. coli* colonization in piglets; however, the metabolites responsible for this protective effect remain unidentified. Here, using a bioactivity-guided fractionation approach, we identify C17 fengycin B as a major contributing metabolite associated with the anti-ETEC activity of DB9011 and show enhanced growth inhibition during co-treatment with colistin. These findings clarify the functional contribution of *Bacillus*-derived metabolites to pathogen inhibition and establish a mechanistic basis for strain-specific antimicrobial activity.

## INTRODUCTION

Global population growth continues to drive a substantial rise in demand for animal protein (1). While interest in alternative protein sources is increasing, technological limitations currently limit their full replacement of livestock-derived proteins at scale (2). Consequently, reliance on intensified production systems persists, often exposing animals to multiple stressors that impair health and productivity (3). Particularly, in pig farming, overcrowding, regrouping, poor sanitation, and abrupt weaning increase susceptibility to enteric infections (4). Post-weaning diarrhea (PWD) is one of the most serious health problems in pig production, primarily caused by enterotoxigenic *Escherichia coli* (ETEC) (5). PWD leads to significant economic losses worldwide due to high morbidity and mortality rates, veterinary treatment costs, and impaired growth (6). Clinical symptoms include watery diarrhea, dehydration, loss of appetite, and lethargy, which severely compromise piglet welfare and productivity (7).

To control PWD and other ETEC-associated enteric diseases, antibiotics—particularly orally administered prophylactic and metaphylactic treatments—have historically been widely used in pig production (8). However, concerns regarding antimicrobial resistance (AMR) have driven major regulatory and policy shifts worldwide. Notably, the European Union banned the use of antimicrobials as growth promoters in 2006 (9), and veterinary antimicrobial sales subsequently declined substantially across EU member states between 2011 and 2020 (10). Furthermore, surveillance studies have indicated that patterns of antibiotic use in pig production vary considerably across regions, with differences in prophylactic group treatment practices, antimicrobial classes, and administration routes (11–13). In several regions, prophylactic use remains part of routine management, reflecting ongoing regional variability in the implementation of antimicrobial stewardship measures (14, 15). Together, these observations underscore the need for practical and sustainable approaches that can complement existing disease control strategies while maintaining effective management of ETEC-associated disease in pig production (5, 16–18).

Among the proposed alternatives, probiotics have attracted increasing attention (18). Several studies have demonstrated that probiotics can enhance intestinal health, reduce pathogen colonization, and mitigate diarrhea in piglets (19–24). *Bacillus* species are particularly promising as feed probiotics because they produce stress-resistant endospores, enabling survival during feed processing, storage, and passage through the gastrointestinal tract (25, 26). Feeding trials in pigs have shown that dietary supplementation with *Bacillus* improves growth performance, reduces diarrhea, and suppresses infections caused by pathogenic *E. coli* (27–31). The mechanisms underlying these protective effects have been extensively investigated and are understood to involve several processes, including competitive exclusion of pathogens (20), stimulation of the host innate immune system (32, 33), and the production of antimicrobial metabolites (34–37). Particular attention has been directed toward antimicrobial metabolites produced by *Bacillus* spp., which include lantibiotics, polyketides, and lipopeptides (36–38). Although several studies have examined the antibacterial effects of lipopeptides such as fengycins against gram-negative bacteria (39, 40), the identification of strain-specific antimicrobial metabolites that mediate inhibition of enterotoxigenic *E. coli* remains incomplete. This knowledge gap limits our understanding of how *Bacillus*-based probiotics exert protective effects in pathogen-specific contexts.

Based on previous studies demonstrating that *Bacillus subtilis* DB9011 suppresses pathogenic *E. coli* infection in a piglet STEC infection model (31), we sought to identify the extracellular metabolite(s) associated with the anti-ETEC activity of this probiotic strain. To this end, we employed a bioactivity-guided fractionation strategy in which fractions were selected solely on the basis of antibacterial activity against ETEC, followed by mass spectrometric analysis of the most active fractions. Using this approach, we identified C17 fengycin B as a candidate metabolite contributing to the observed antibacterial phenotype. Our findings clarify the contribution of *Bacillus*-derived lipopeptides to ETEC growth inhibition and provide a mechanistic basis for understanding strain-specific antibacterial activity in probiotic-relevant *Bacillus* strains.

## RESULTS

### Antibacterial activity of *B. subtilis* DB9011 culture supernatant

**Fig 1:**
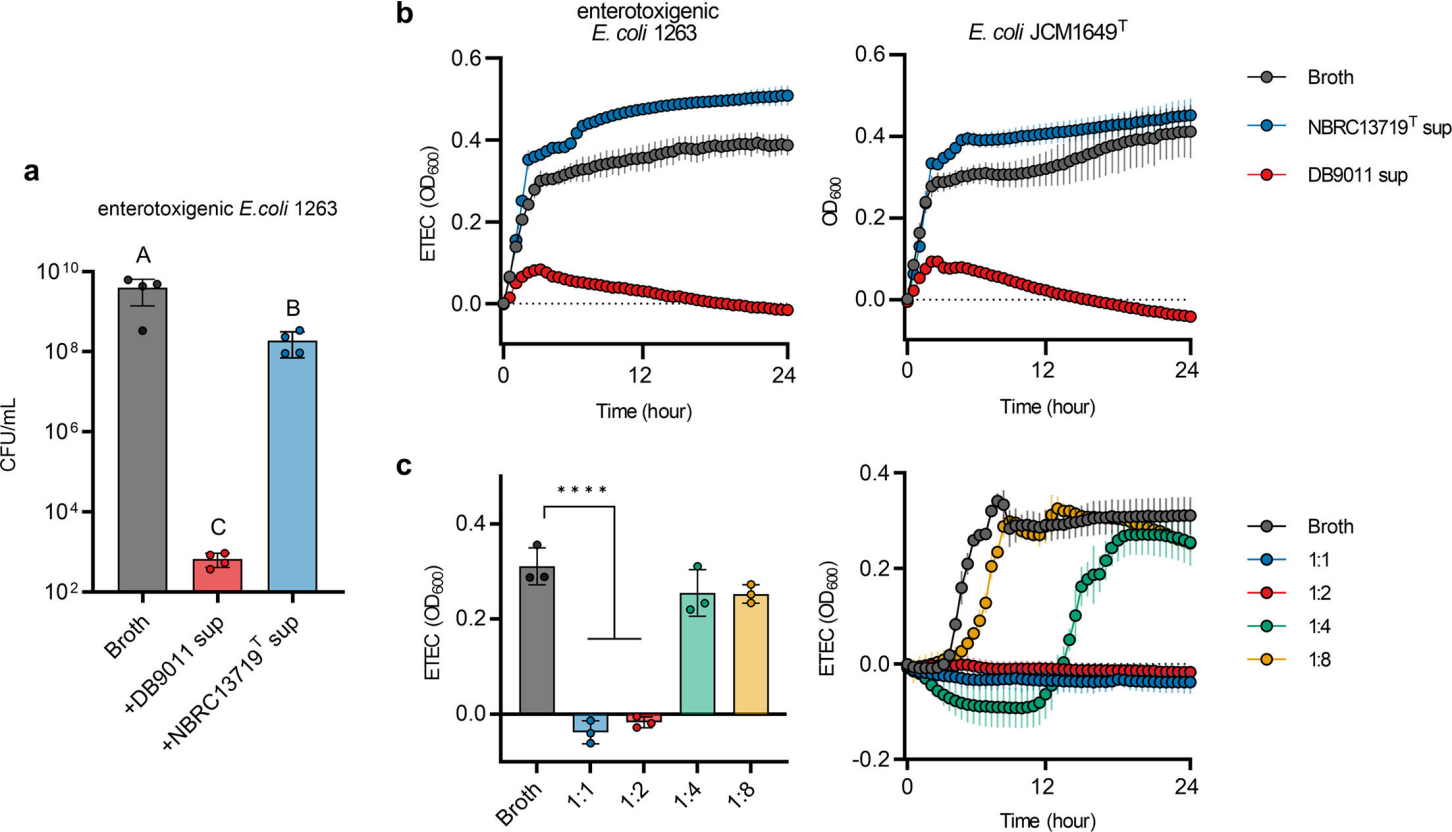
Antibacterial activity of *B. subtilis* DB9011 culture supernatant. (**a**) Viability of enterotoxigenic *E. coli* (ETEC1263) after incubation with the culture supernatant of *B. subtilis* DB9011 and type strain NBRC13719^T^. ETEC1263 cells were cultured for 24 h in the presence of culture supernatant, and viable bacteria were quantified by CFU enumeration. (**b**) Growth inhibition of ETEC1263 and type strain *E. coli* JCM1649^T^ by culture supernatants from *B. subtilis* DB9011 and type strain NBRC13719ᵀ. Pathogenic strains were incubated with an equal volume of culture supernatant, and bacterial growth was monitored for 24 h. (**c**) Concentration-dependent growth inhibition of ETEC1263 by serially diluted DB9011 culture supernatant. Supernatant-to-medium ratios were 1:1, 1:2, 1:4, and 1:8 (vol/vol). Bacterial growth was quantified by measuring the OD_600_ every 30 min for 25 h. Bar graphs represent OD_600_ values of ETEC cultures after 24 h of incubation. Data represent the mean ± SD of three independent experiments. Different letters indicate significant differences (*P* < 0.05). (*P < 0.05; **P < 0.01; ***P < 0.001, one-way ANOVA followed by Tukey’s or Dunnett’s multiple comparison test).

The antibacterial activity of *B. subtilis* DB9011 against enterotoxigenic *E. coli* (ETEC1263) was evaluated by quantifying viable cell counts (colony-forming units [CFUs]) after 24-h incubation in the presence of culture supernatant. The DB9011 supernatant significantly reduced ETEC CFU compared with both the medium control and the NBRC13719ᵀ supernatant (Fig. 1a), resulting in an approximately 6.6-log reduction relative to the medium control and indicating a marked decrease in the number of culturable ETEC cells. Although the NBRC13719ᵀ supernatant also reduced CFU, its effect was substantially weaker, corresponding to an approximately 1.2-log reduction relative to the medium control and indicating a strain-dependent difference in antibacterial activity. Similar results were observed in the 24-h growth assay. The NBRC13719ᵀ supernatant caused only limited growth suppression, whereas the DB9011 supernatant markedly suppressed the increase in OD values of both ETEC1263 and the *E. coli* type strain JCM1649ᵀ during incubation (Fig. 1b). DB9011 supernatant was then mixed with ETEC1263 culture at ratios ranging from 1:1 to 1:8 (vol/vol). The antibacterial effect decreased in a concentration-dependent manner (Fig. 1c), with the strongest suppression at the 1:1 ratio and progressively weaker effects at higher dilutions. These findings indicate that *B. subtilis* DB9011 secretes antibacterial factor(s) that suppress ETEC growth in a concentration-dependent manner.

**Fig 2:**
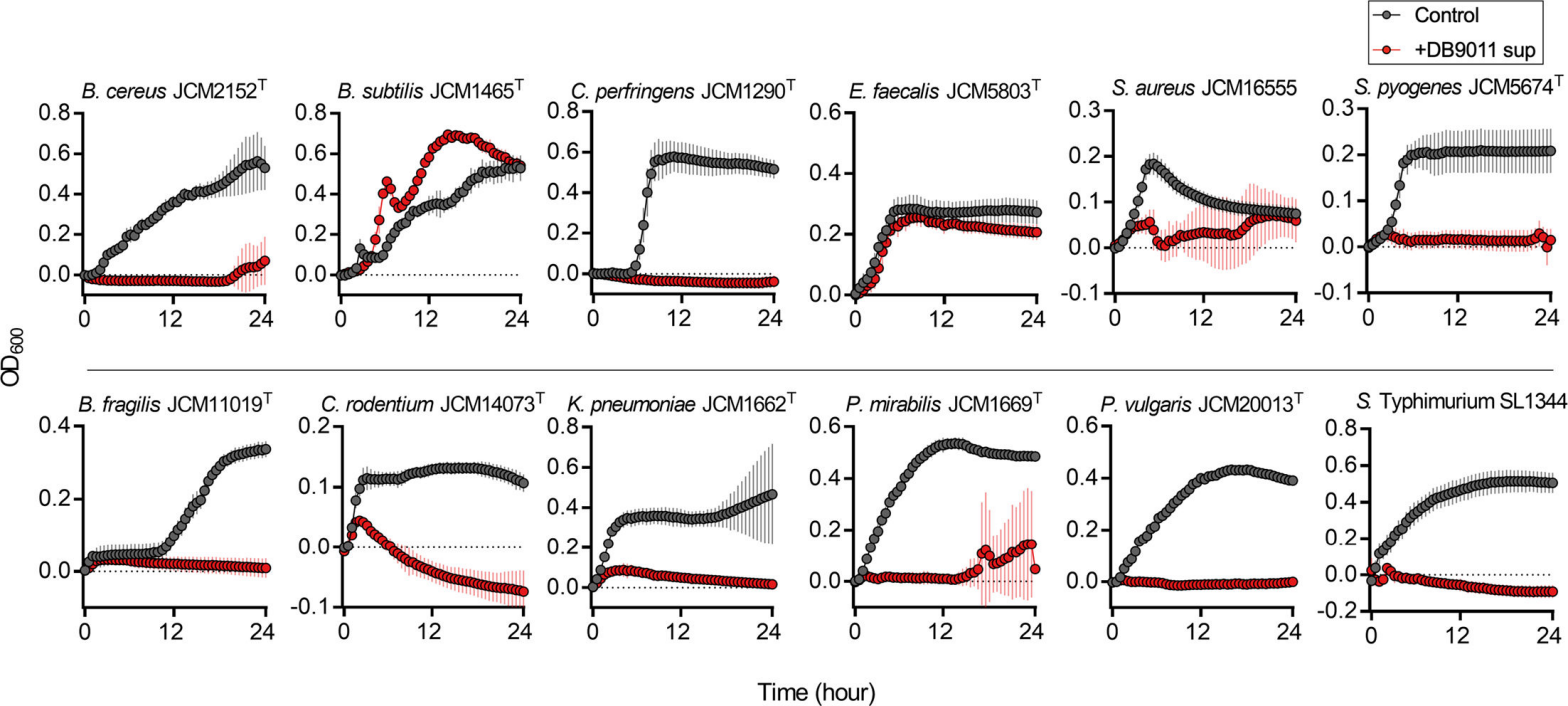
Antibacterial activity of DB9011 culture supernatant against diverse bacterial species. Pathogenic strains were incubated with an equal volume of culture supernatant, and bacterial growth was monitored for 24 h by measuring OD_600_. The upper panels show gram-positive bacteria, and the lower panels show gram-negative bacteria. Control (black circles) and DB9011 supernatant-treated samples (red circles) are shown. Data are presented as mean ± SD (*n* = 3).

To determine whether the inhibitory effect observed against ETEC represented a strain-specific or broader antimicrobial capacity, DB9011 supernatant was tested against a wider range of bacterial species. It significantly suppressed the growth of various gram-negative and gram-positive pathogens, including *Bacillus cereus* JCM2152^T^, *Clostridium perfringens* JCM1290^T^, *Staphylococcus aureus* JCM16555, *Streptococcus pyogenes* JCM5674^T^, *Bacteroides fragilis* JCM11019^T^, *Citrobacter rodentium* JCM14703^T^, *Klebsiella pneumoniae* JCM1662^T^, *Proteus mirabilis* JCM1669^T^, *Proteus vulgaris* JCM20013^T^, and *Salmonella* Typhimurium SL1344, while showing no effect on *B. subtilis* type strain JCM1465^T^ and *Enterococcus faecalis* JCM5803^T^ (Fig. 2). These results suggest that *B. subtilis* DB9011 secretes antibacterial compound(s) with broad-spectrum activity against diverse (opportunistic) pathogens, but not against *B. subtilis* and *E. faecalis*.

### Purification of antibacterial components

**Fig 3:**
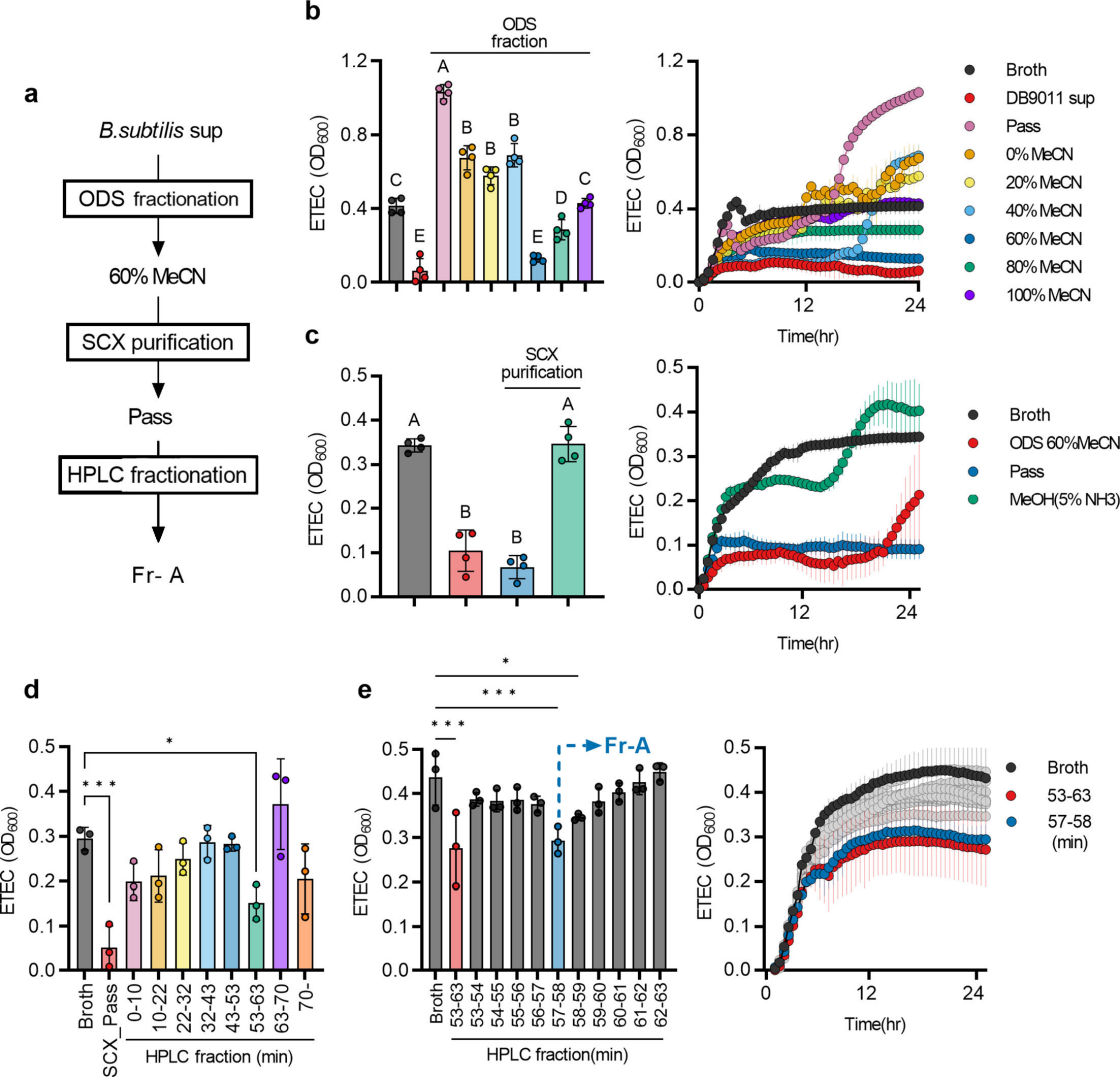
Stepwise purification of antibacterial components from *B. subtilis* DB9011 culture supernatant. (**a**) Schematic overview of the purification procedure. The culture supernatant was subjected to reversed-phase ODS chromatography, SCX purification, and ODS-HPLC fractionation to isolate active compounds. (**b**) Antibacterial activity of ODS fractions eluted with increasing concentrations of MeCN. The 60% MeCN fraction showed the strongest inhibition of ETEC growth. The right panel shows representative growth curves over 24 h. (**c**) SCX purification of the 60% MeCN fraction. Antibacterial activity was assessed for the flow-through and 5% ammonia–methanol eluate. The flow-through fraction retained strong activity. (**d**) Evaluation of ODS-HPLC fractions eluted at 2-min intervals. Fractions eluted at 53–63 min markedly suppressed ETEC growth. (**e**) Fine mapping of the active window identified the 57- to 58-min subfraction (designated Fr-A) as the most potent. Growth curves of ETEC treated with Fr-A or adjacent fractions are shown (right panel). Bar graphs represent OD_600_ values of ETEC cultures after 24 h of incubation. Data are shown as the mean ± SD of three independent experiments. Different letters indicate significant differences (*P* < 0.05). (*P < 0.05; **P < 0.01; ***P < 0.001, one-way ANOVA followed by Tukey’s or Dunnett’s multiple comparison test).

To isolate and identify the antibacterial component(s) present in the culture supernatant of *B. subtilis* DB9011, a stepwise purification procedure was performed (Fig. 3a). The process comprised three main steps: octadecylsilyl (ODS) reversed-phase chromatography, strong cation exchange (SCX) purification, and preparative ODS-high-performance liquid chromatographic (HPLC) fractionation. In the first step, the culture supernatant was subjected to ODS column chromatography and eluted with increasing concentrations of acetonitrile (MeCN), yielding seven fractions. Antibacterial activity was detected across multiple ODS fractions, with activity predominantly associated with the 60% MeCN-eluted fraction, which exhibited the strongest inhibitory effect against ETEC1263 compared with the broth control (Fig. 3b; Fig. S1a).

To further characterize the antimicrobial component(s) enriched in the 60% MeCN fraction, the active material was subjected to SCX chromatography. The flow-through fraction, but not the 5% ammonia–methanol eluate, retained strong antibacterial activity comparable to that of the original 60% MeCN extract (Fig. 3c). A similar observation was made in strong anion exchange (SAX) purification (Fig. S1b), supporting the notion that the active component(s) is/are nonionic under the test conditions used. The SCX flow-through fraction was subsequently fractionated using ODS-HPLC, guided by the chromatographic elution profile (Fig. S1c). Antibacterial assays revealed that fractions eluting between 53 and 63 min markedly suppressed ETEC1263 growth (Fig. 3d; Fig. S1d). Earlier fractions (0–10 min and 10–22 min) exhibited slight apparent activity, but these effects were not statistically significant, and the 0- to 10-min region showed unresolved peak patterns (Fig. S1c). Therefore, the 53- to 63-min window was prioritized for further assays. The subfraction eluting at 57–58 min displayed the most potent inhibitory effect and was designated as fraction A (Fr-A). Fr-A consistently inhibited ETEC1263 growth over a 24-h incubation period (Fig. 3e). Next, the antimicrobial activity of the ODS, SCX, and HPLC fractions against *S*. Typhimurium was evaluated. Consistent with the results for ETEC1263, Fr-A exhibited the strongest inhibitory activity (Fig. S2a through d). These findings indicate that Fr-A contained the principal antibacterial metabolite(s) produced by DB9011. Hence, Fr-A was selected for structural characterization through liquid chromatography–quadrupole time-of-flight mass spectrometry (LC-QTOF/MS).

### Identification of antibacterial components using LC-QTOF/MS

**Fig 4:**
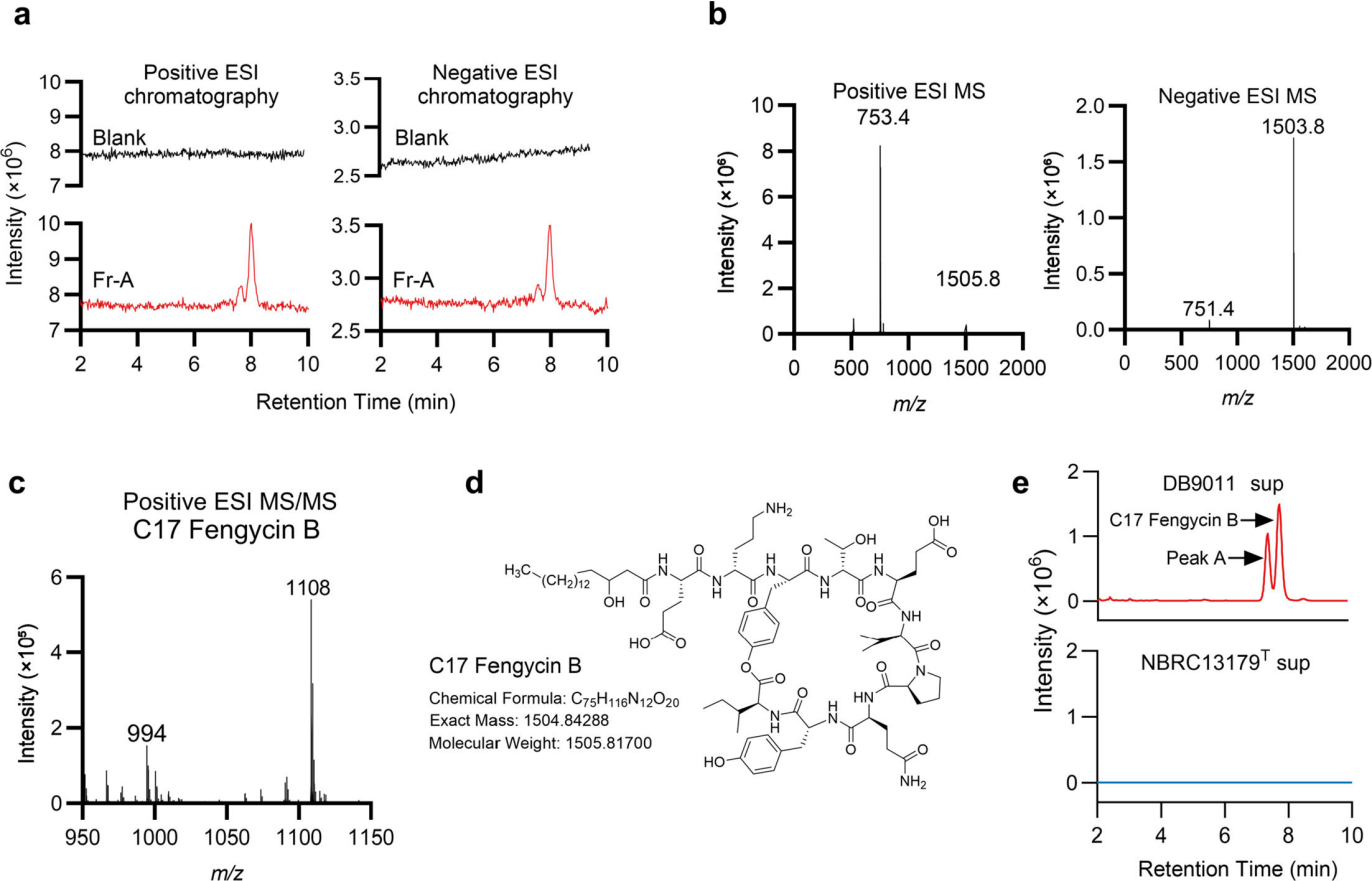
Identification of C17 fengycin B as the antibacterial component in *B. subtilis* DB9011 culture supernatant. (**a**) LC chromatograms of Fr-A analyzed using LC-QTOF/MS under positive and negative ESI modes. (**b**) Mass spectra of Fr-A showing the detection of ions at m/z 753.4 ([M  +  2H]²^+^) and m/z 1,505.8 ([M  +  H]^+^) in positive ESI mode, and m/z 1,503.8 ([M – H]⁻) in negative ESI mode. (**c**) ESI-MS/MS spectrum of the precursor ion m/z 1505.8 ([M  +  H]^+^), revealing major fragment ions at m/z 1,108 and 994, corresponding to known fragmentation patterns of fengycin B. (**d**) Proposed chemical structure of C17 fengycin B with molecular formula C_73_H_116_ N_12_O_20_ (exact mass, 1504.54). (**e**) Extracted ion chromatograms of m/z 753.4 ([M  +  2H]²^+^) comparing *B. subtilis* DB9011 and the type strain *B. subtilis* NBRC13719ᵀ. The signal corresponding to C17 fengycin B was detected only in DB9011. Data are representative of three independent experiments.

To elucidate the identity of the antibacterial component enriched in Fr-A, we performed high-resolution MS analysis using LC-QTOF/MS, which revealed a prominent peak in both positive and negative electrospray ionization (ESI) modes (Fig. 4a). In positive ESI mode, the mass spectrum showed major ions at *m/z* 753.4 ([M  +  2H]²^+^) and *m/z* 1,505.8 ([M  +  H]^+^), whereas in negative ESI mode, a corresponding ion at *m/z* 1,503.8 ([M – H]⁻) was detected (Fig. 4b), indicating a molecular weight of approximately 1,505 Da. To further characterize this molecule, MS/MS analysis was performed using the *m/z* 1,505.8 ion as the precursor. The resulting fragment ions at m/z 1,108 and 994 corresponded to the sequential loss of fatty acid-Glu (–398 Da) and fatty acid-Glu-Orn (–512 Da), respectively, from the N-terminal portion of fengycin B (41–44). Based on these fragmentation patterns and the exact mass, the compound was identified as C17 fengycin B (Fig. 4c and d; Table S1), a cyclic lipopeptide belonging to the fengycin family of *Bacillus*-derived secondary metabolites, previously characterized for antifungal activity (44). Notably, comparative LC-MS analysis demonstrated that C17 fengycin B was absent in the culture supernatant of *B. subtilis* NBRC13719ᵀ, which lacked antibacterial activity (Fig. 4e). Furthermore, another peak detected in Fig. 4e, designated as Peak A, appeared as a minor peak in Fr-A. The same peak was more abundant in the 56- to 57-min subfraction (Fig. S3a); however, this 56- to 57-min subfraction exhibited no antibacterial activity against ETEC 1263 (Fig. 3e; Fig. S3b). These findings indicate that C17 fengycin B production is associated with the antibacterial activity of *B. subtilis* DB9011 and likely contributes to its growth-inhibitory effects on ETEC1263.

### Functional validation of Fr-A as a key contributor to DB9011-mediated inhibition of ETEC

LC–MS analysis revealed that Fr-A (Fig. 3e) contained C17 fengycin B at an approximately fivefold higher concentration than that detected in the original culture supernatant (Table S2). To determine whether this fengycin-enriched fraction contributes to the antibacterial phenotype of DB9011, we evaluated its concentration-dependent activity and assessed its effect when added to a non-inhibitory NBRC13719ᵀ culture supernatant.

**Fig 5:**
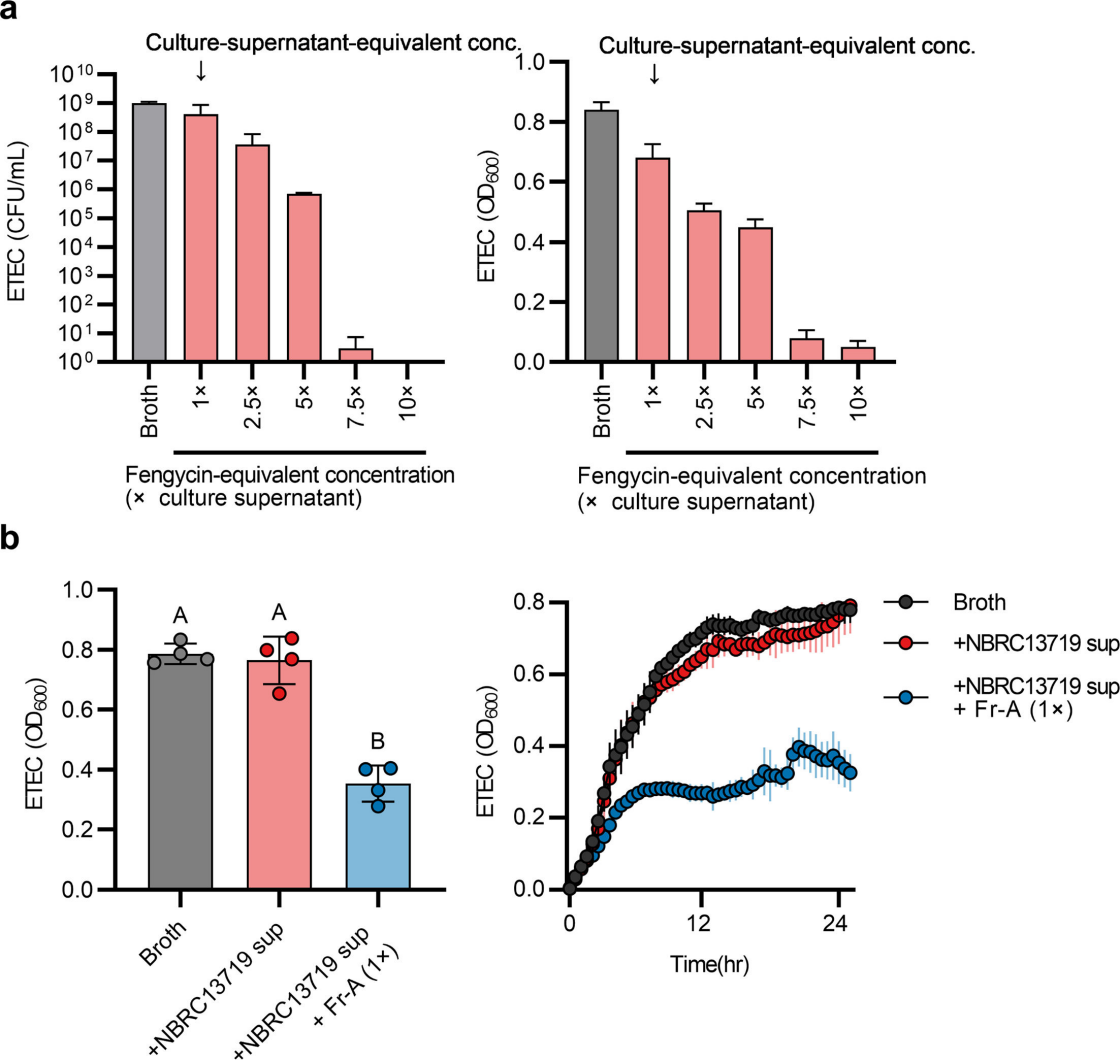
Concentration-dependent antibacterial activity of the fengycin-containing fraction (Fr-A). (**a**) Concentration-dependent activity of Fr-A. (Left) Viable cell counts (CFU/mL) of ETEC cultured in the presence of Fr-A adjusted to 1× (equivalent to the concentration of C17 fengycin B detected in the original *B. subtilis* DB9011 culture supernatant), 5×, or 10×. (Right) Corresponding OD_600_ measurements under the same concentration series. Growth inhibition increased with increasing concentration, consistent with the CFU results. (**b**) Functional complementation of a noninhibitory culture supernatant. OD_600_ values after 24 h of ETEC cultivation in broth alone, NBRC13719ᵀ culture supernatant, or NBRC13719ᵀ supernatant supplemented with Fr-A at 1×. The right panel shows representative growth curves over 24 h. Supplementation with Fr-A partially restored inhibitory activity, whereas Fr-A alone or NBRC13719ᵀ supernatant alone did not markedly suppress growth.

Both optical density measurements and CFU enumeration demonstrated a clear concentration-dependent antibacterial effect. At the culture-supernatant–equivalent concentration (1×), OD values were reduced and viable cells remained detectable, indicating a predominantly bacteriostatic effect. Increasing the concentration to 5× further reduced viable cell numbers by approximately 2.8 log compared with 1×, although viable cells were still detectable. In contrast, a 10× concentrated preparation markedly suppressed growth and reduced CFU counts to below the detection limit, consistent with bactericidal activity at higher concentrations (Fig. 5a). Furthermore, supplementation of the non-inhibitory NBRC13719ᵀ culture supernatant with Fr-A at a concentration equivalent to that present in the DB9011 supernatant resulted in reduced ETEC growth, as assessed by OD measurements (Fig. 5b), whereas neither Fr-A nor the NBRC13719ᵀ supernatant alone suppressed growth. Together, these findings indicate that the fengycin-containing fraction functionally contributes to the antibacterial activity exhibited by *B. subtilis* DB9011.

### Membrane-associated effects of Fr-A and combined treatment with colistin

Fengycin has been reported to perturb fungal membranes through pore formation (45), and membrane-associated effects have also been described in gram-negative bacteria (39). Based on these reports, we examined whether the fengycin-containing fraction (Fr-A) affects membrane integrity in ETEC.

**Fig 6:**
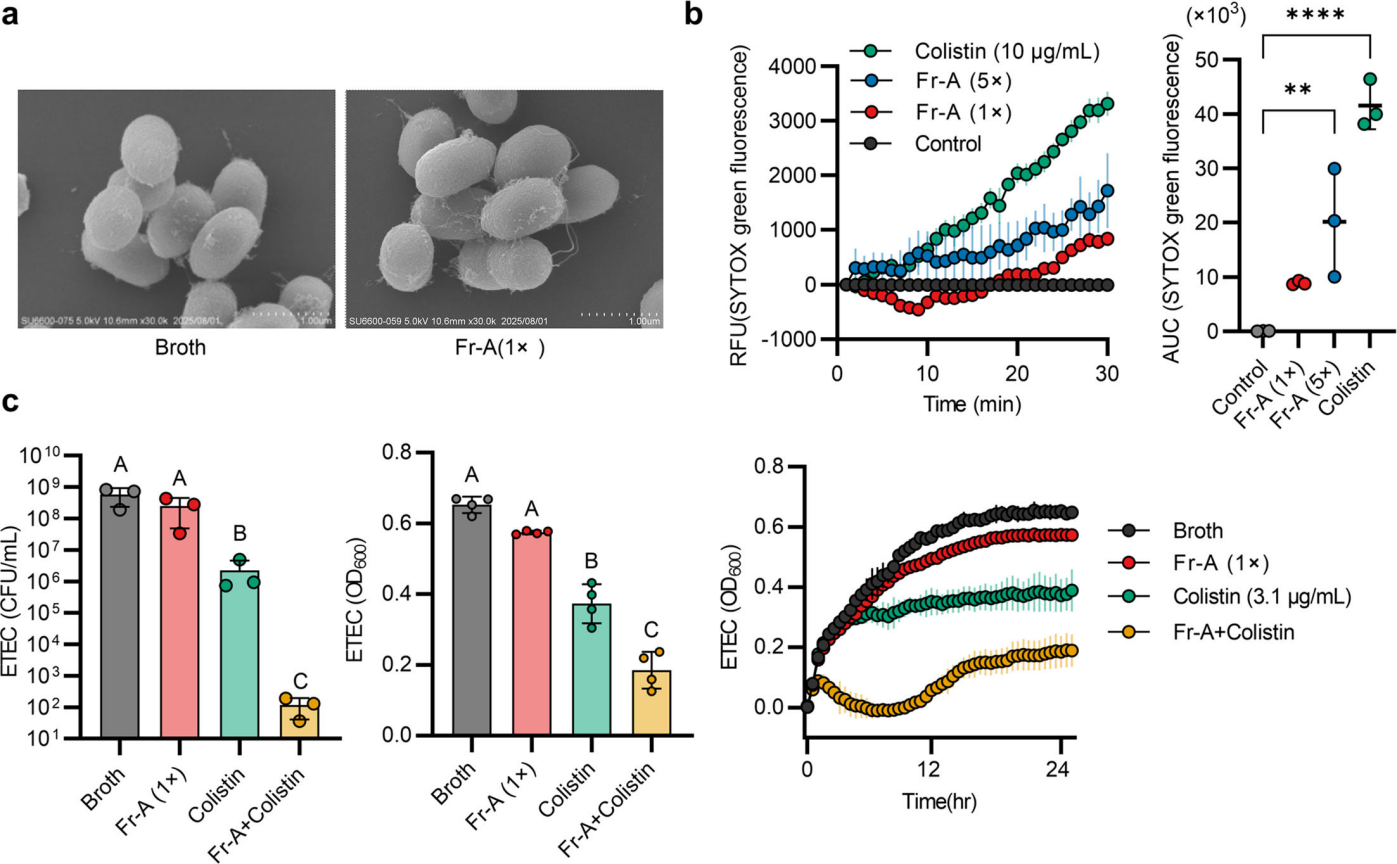
Combined effects of Fr-A and colistin on ETEC viability. (**a**) Scanning electron microscopy (SEM) images of ETEC cells cultured in basal LB broth with or without Fr-A (1×). No overt morphological disruption was observed under the conditions tested. (**b**) SYTOX Green uptake as an indicator of membrane permeabilization. (Left) Time-dependent SYTOX Green fluorescence (RFU) measured over 30 min in cultures treated with Fr-A at 1× or 5× (relative to the concentration of C17 fengycin B present in the original DB9011 culture supernatant), colistin (10 µg/mL), or untreated control. (Right) AUC values calculated from fluorescence profiles. Fr-A induced a moderate but reproducible increase in membrane permeability, whereas colistin produced the highest uptake. (**c**) Combined effects of Fr-A and colistin on ETEC growth. Cultures were treated with Fr-A (1×), colistin (3.1 µg/mL), or both compounds. (Left) CFU after 24-h incubation. (Center) OD₆₀₀ after 24 h. (Right) Representative OD_600_-based growth curves over 24 h. The combination treatment resulted in significantly greater growth inhibition than either compound alone under the conditions tested. Data represent mean ± SD from three independent experiments. Different letters indicate significant differences (*P* < 0.05). (*P < 0.05; **P < 0.01; ***P < 0.001, one-way ANOVA followed by Tukey’s or Dunnett’s multiple comparison test).

SEM analysis revealed no overt morphological disruption in Fr-A–treated cells compared with untreated controls (Fig. 6a). To assess membrane permeability, SYTOX Green uptake assays were performed. Fr-A increased SYTOX Green fluorescence relative to the untreated control in a concentration-dependent manner (1× and 5×; Fig. 6b). Quantification of the area under the curve (AUC) showed that SYTOX Green fluorescence was significantly increased at 5× compared with the untreated control (*P <* 0.05), whereas the 1× treatment did not reach statistical significance. The magnitude of fluorescence remained lower than that observed with colistin (10 µg/mL), but the 5× increase was reproducible across independent experiments. These results indicate that Fr-A induces measurable changes in membrane permeability without causing gross structural damage.

To examine whether the Fr-A-induced increase in membrane permeability could influence susceptibility to a membrane-targeting agent, we tested its combined effect with a subinhibitory concentration of colistin, a well-characterized antibiotic active against gram-negative bacteria. Co-treatment with Fr-A (1×) and a subinhibitory concentration of colistin (3.1 µg/mL) resulted in significantly greater growth inhibition than either treatment alone, as reflected by reduced CFU counts and sustained suppression of OD-based growth (Fig. 6c). These findings are consistent with a complementary interaction between Fr-A and colistin under the conditions tested.

## DISCUSSION

In this study, we employed a bioactivity-guided screening strategy using enterotoxigenic *E. coli* as an indicator organism and identified C17 fengycin B as a principal antimicrobial metabolite produced by *B. subtilis* DB9011. Fractions enriched in C17 fengycin B inhibited ETEC1263 and suppressed *S*. Typhimurium SL1344 (Fig. 3e; Fig. S2), indicating activity against gram-negative enteric pathogens.

As culture supernatants were applied at a 1:1 ratio in the initial screening assays, nutrient dilution could theoretically have contributed to growth suppression. However, supernatant from the type strain *B. subtilis* NBRC13719ᵀ, prepared under identical conditions, did not inhibit pathogen growth. Furthermore, CFU enumeration after 24 h confirmed that only the DB9011 supernatant significantly reduced viable ETEC counts, rather than merely delaying proliferation (Fig. 1a). The pH values of the spent media were similar (DB9011, pH 6.45; NBRC13719ᵀ, pH 6.60; medium control, pH 6.70), indicating that differential pH is unlikely to account for the strain-specific inhibitory effect. Together, these observations demonstrate that the inhibitory phenotype cannot be attributed solely to nutrient limitation and is instead mediated by DB9011-derived bioactive metabolites. The antimicrobial activity of DB9011 was clearly strain-specific. Whereas the DB9011 supernatant inhibited multiple gram-negative pathogens, NBRC13719ᵀ exhibited no detectable activity. Such variability is consistent with previous reports describing substantial diversity in lipopeptide biosynthesis and antimicrobial spectra among *Bacillus* strains (46, 47), reflecting differences in secondary metabolite production profiles. Notably, DB9011 did not inhibit *E. faecalis*, in agreement with earlier observations that *B. subtilis* culture supernatants generally lack activity against this species growth(48).

Although fengycins have long been recognized as antifungal metabolites (44) and inhibitors of gram-positive bacteria such as *S. aureus* and *E. faecalis* (41, 48), more recent studies have demonstrated antibacterial activity against gram-negative organisms, including *E. coli* and *Salmonella*, through membrane-associated mechanisms (39, 40). Medeot et al. (39) reported that fengycin-containing fractions from *Bacillus amyloliquefaciens* MEP218 induced membrane-associated alterations in gram-negative bacteria, including surface changes detected by atomic force microscopy and potassium ion efflux. However, these studies did not establish whether fengycin production by probiotic-relevant *Bacillus* strains is sufficient to explain pathogen inhibition, nor did they define fengycin as a determinant of strain-specific antimicrobial phenotypes. Thus, the contribution of fengycin production to the suppression of enterotoxigenic *E. coli* by candidate probiotic strains remained unresolved.

Importantly, inhibition of ETEC1263 by C17 fengycin B was observed only at concentrations exceeding those present in the native culture supernatant, indicating that fengycin B alone is unlikely to fully account for the antibacterial activity of the native DB9011 supernatant. Bacteriostatic effects were observed at up to fivefold the supernatant-equivalent concentration, whereas bactericidal activity required a substantially higher (10-fold) concentration (Fig. 5a). These findings indicate that C17 fengycin B, at its physiological concentration in the DB9011 supernatant, is unlikely to fully account for the overall antibacterial activity observed. Previous studies have shown that combinations of surfactins and fengycins exhibit enhanced antimicrobial activity relative to individual compounds (49, 50), suggesting cooperative or additive interactions among lipopeptides. However, the *in vivo* production levels of these metabolites may differ from those observed *in vitro*, and higher production of fengycin under intestinal conditions could potentially influence the relative contribution of individual compounds. Consistent with this notion, HPLC fractionation of the SCX flow-through revealed an active window spanning 53–63 min, within which the 56- to 57-min fraction containing C17 fengycin B displayed the strongest activity. However, this activity did not reach that of the unfractionated SCX sample, indicating that additional antibacterial metabolites are present within this window and likely contribute to the complete inhibitory phenotype.

Fengycins have been widely reported to interact with biological membranes across diverse systems (51–58). Biophysical analyses have demonstrated pore formation in planar lipid bilayers (45, 56, 57). In gram-negative bacteria, membrane-associated alterations—including changes in surface topography and potassium ion release—have been documented following exposure to fengycin-containing fractions (39). Moreover, studies using model phospholipid bilayers have shown that fengycin interacts with membrane lipids and perturbs membrane organization (58). In agreement with these reports, our SYTOX Green assay demonstrated increased membrane permeability in ETEC1263 following treatment with fengycin-containing fractions, although the magnitude of permeabilization was lower than that observed with colistin (Fig. 6b). SEM analysis, in contrast, did not reveal overt membrane rupture or extensive structural disruption, suggesting that fengycin B modulates membrane permeability without inducing catastrophic membrane damage. Collectively, these observations support membrane permeability modulation as a conserved feature of fengycin activity.

Accumulating evidence indicates that membrane-active peptides may exert biological effects beyond direct membrane disruption. For instance, C17 fengycin B at low concentrations induced metacaspase-dependent apoptosis in *Fusarium oxysporum* despite causing minimal membrane damage (59). Similarly, the bacteriocin nisin inhibits bacterial growth not only through pore formation but also via binding to lipid II and interference with cell wall biosynthesis (60). These findings highlight the influence of membrane-active lipopeptides on essential cellular processes beyond membrane permeabilization. Although the DB9011 supernatant did not inhibit *E. faecalis* growth, surfactins and fengycins have been reported to interfere with quorum-sensing in this species (48), raising the possibility that DB9011-derived metabolites may modulate bacterial physiology even in the absence of overt bactericidal activity.

From an applied perspective, colistin remains widely used for the treatment of ETEC infections in livestock production (61, 62). Strategies that reduce reliance on critically important antibiotics are therefore of interest within a One Health framework (63). In the present study, co-treatment with fengycin B and a subinhibitory concentration of colistin resulted in greater growth inhibition than either compound alone under the conditions tested (Fig. 6c). As formal synergy analyses (e.g., checkerboard assays or fractional inhibitory concentration index determinations) were not performed, the interaction cannot be definitively classified as synergistic. Nevertheless, the enhanced inhibitory effect observed during co-treatment is consistent with a complementary or additive interaction between the two agents.

In conclusion, our bioactivity-guided approach identified C17 fengycin B as a major contributing metabolite associated with the antibacterial phenotype of *B. subtilis* DB9011 and demonstrated its contribution to the suppression of enterotoxigenic *E. coli* and *S*. Typhimurium. However, the concentration of fengycin present in the native supernatant is unlikely to fully explain the observed antibacterial activity, indicating that additional metabolites participate in the inhibitory phenotype. In addition, the physicochemical properties of the active metabolites, including thermal stability, pH sensitivity, and factors governing their production over time, were not systematically examined in this study. Future work should address these aspects and evaluate the *in vivo* production dynamics, stability, and functional relevance of DB9011-derived metabolites within the intestinal environment and animal infection models.

## MATERIALS AND METHODS

### Bacterial strains and culture conditions

*Bacillus subtilis* DB9011, a probiotic candidate strain previously shown to protect piglets in an STEC infection model, was provided by SDS Biotech K.K. (Tokyo, Japan) (31). The type strain *B. subtilis* NBRC13719ᵀ, which consistently lacked antibacterial activity against ETEC and other tested pathogens in our assays, was obtained from the National Institute of Technology and Evaluation (NITE, Tokyo, Japan) and was used as a non-inhibitory reference strain for comparative analyses. Both strains were grown in Luria–Bertani (LB) broth (BD Difco) at 30°C with agitation at 180 rpm for 30 h. Following incubation, the cultures were centrifuged at 10,000 × *g* for 30 min at 4°C, and the supernatants were passed through 0.22-μm pore-size polyethersulfone filters (Merck Millipore) to obtain sterile cell-free culture supernatants.

The enterotoxigenic *E. coli* strain 1263 (ETEC1263) was isolated from diarrheic feces of piglets raised at a commercial swine farm in Miyagi, Japan. *S*. Typhimurium SL1344 was generously provided by Dr. Nobuhiko Okada (Kitasato University, Tokyo, Japan). *E. coli* JCM1649ᵀ, *K. pneumoniae* JCM1662ᵀ, *B. fragilis* JCM11019ᵀ, *C. perfringens* JCM1290ᵀ, *C. rodentium* JCM14703ᵀ, *B. cereus* JCM2152ᵀ, *P. mirabilis* JCM1669ᵀ, *P. vulgaris* JCM20013ᵀ, *S. pyogenes* JCM5674ᵀ, and *S. aureus* JCM16555 were obtained from the Japan Collection of Microorganisms (JCM, RIKEN Bioresource Center, Tokyo, Japan).

### Antibacterial screening and bioactivity-guided assays

For broad-spectrum antibacterial screening, all bacterial strains were pre-cultured in GAM broth (Nissui Pharmaceutical Co., Tokyo, Japan) at 37°C under anaerobic conditions using AnaeroPack systems (Mitsubishi Gas Chemical Co., Tokyo, Japan). For bioactivity-guided metabolite identification, ETEC1263 and S. Typhimurium SL1344 were pre-cultured in LB broth at 37°C with shaking at 180 rpm for 24 h. Precultures were diluted 1:100 into 3 mL of fresh GAM or LB broth and incubated for 1–3 h under the same conditions until reaching early logarithmic phase. Bacterial suspensions (100 μL) were then dispensed into sterile flat-bottom 96-well microplates (Corning Inc., Corning, NY, USA).

#### Culture supernatant assays

Cell-free culture supernatants of *B. subtilis* strains were prepared by centrifugation and filtration and subsequently diluted 1:2, 1:4, or 1:8 (vol/vol) with fresh medium. Equal volumes (75 μL each) of bacterial suspension and undiluted or diluted supernatants were mixed in 96-well plates, resulting in a final assay volume of 150 μL per well. Control wells received fresh medium instead of supernatant.

#### Fraction assays

For chromatographic fraction assays, 10 μL aliquots of each fraction were dispensed into 96-well plates and air-dried completely at room temperature to remove solvent and concentrate the sample. After drying, 150 μL of early-log-phase bacterial suspension was added to each well to reconstitute the fraction.

#### Colistin co-treatment assays

Colistin sulfate (FUJIFILM Wako Pure Chemical Corporation, Japan) was used in combination experiments. A sub-inhibitory concentration corresponding to the MIC_50_ (3.1 μg/mL for ETEC1263 under our assay conditions) was added at the start of incubation to evaluate combinatorial effects.

Plates for broad-spectrum screening were incubated anaerobically at 37°C for 48 h in an anaerobic chamber (COY Laboratory Products, Grass Lake, MI, USA) with an atmosphere of 90% N₂, 5% H₂, and 5% CO₂ (corresponding to Fig. 2). Plates for bioactivity-guided metabolite identification were incubated aerobically at 37°C for 48 h (ETEC1263 and *S.* Typhimurium assays). Bacterial growth was monitored by measuring OD_600_ at 0.5- to 1-h intervals using a Stratus microplate reader (Cerillo Inc., Charlottesville, VA, USA).

For viable cell quantification, cultures were sampled after 24-h incubation. Serial 10-fold dilutions were prepared in sterile 1× phosphate-buffered saline (PBS), and 50 μL aliquots were spread onto LB agar plates. Plates were incubated at 37°C for at least 24 h, and CFUs were enumerated. CFU counts were calculated as CFU/mL based on dilution factors.

### Purification and fractionation of antibacterial components

#### ODS column fractionation

Approximately 1,200 mL of DB9011 culture supernatant was applied to a DSC-18 SPE column (Merck), and the flow-through was collected. Elution was performed with increasing concentrations of MeCN in water (0%–100% in 20% increments). Each fraction was evaporated to remove organic solvents, reconstituted with distilled water to achieve an equal or fivefold concentration relative to that in the original culture supernatant, and tested using the bacterial growth inhibition assay.

#### SCX purification

The active 60% MeCN ODS fraction (12.5-fold concentrated) was adjusted to 15 mL with distilled water and subjected to SCX column purification (Bond Elut SCX; Agilent Technologies). After conditioning the column with methanol and distilled water, the sample was loaded, and the flow-through was collected. The column was subsequently washed with distilled water and methanol, followed by elution with methanol containing 5% ammonia. All fractions were evaporated to remove solvents, neutralized to pH 7.0 with formic acid or ammonia, reconstituted with distilled water to a fivefold concentration relative to that in the original culture supernatant, and tested using the bacterial growth inhibition assay.

#### Preparative HPLC fractionation

The SCX flow-through fraction was dried, dissolved in 120 μL of methanol, and diluted with 180 μL of distilled water to yield a 40% methanol solution. After filtration through a hydrophilic PTFE syringe filter, the sample was subjected to preparative HPLC (1260 Infinity; Agilent Technologies) using an Atlantis OBD HSS T3 column (10  ×  150  mm, 5 μm; Waters) at a flow rate of 5 mL/min. The mobile phases were distilled water (A) and MeCN (B), with a gradient of 40%–60% B over 70 min. Detection was performed with a diode array detector (DAD) at 220, 254, and 270 nm. Fractions were collected at specified intervals and concentrated to a fivefold concentration relative to that in the original culture supernatant. The active fraction eluted at 53–63 min was further subfractionated in 1-min intervals for detailed analysis.

### LC-QTOF/MS analysis of fengycin

LC-QTOF/MS analysis was performed using an ACQUITY UPLC H-Class system coupled with an Xevo G2-XS QT of mass spectrometer (Waters). Data were acquired and processed with MassLynx V4.1. Samples (1 μL) were injected into an ACQUITY UPLC HSS T3 column (2.1 ×  100  mm, 1.8 μm; Waters) at 0.4 mL/min using a 1:1 mixture of 0.1% formic acid in water and MeCN as the mobile phase. Analyses were performed in both positive and negative ESI modes with the following parameters: scan range, 50-2000 Da; source temperature, 150°C; spray voltage, +3,000  V/–2,500 V; cone gas, 50 psi; desolvation gas, 800 psi; desolvation temperature, 350°C; collision energy, 10-40 V; scan time, 0.2 s. Leucine enkephalin (0.2 mg/L) was used as a lock mass.

### Scanning electron microscopy

Bacterial cells were fixed at 4°C in freshly prepared 2.5% glutaraldehyde in PBS overnight. After washing with PBS, the specimens were transferred into a 0.1 M cacodylate buffer (pH 7.4), treated with 1% tannic acid to improve conductivity, and postfixed in 1% osmium tetroxide in the same buffer at 4°C for 2 h. The samples were dehydrated through a graded ethanol series to absolute ethanol, infiltrated with tert-butyl alcohol, frozen at –10°C, and dried using a vacuum freeze-dryer (VFD-21S; Vacuum Device, Ibaraki, Japan). The dried specimens were mounted on stubs, coated with a thin osmium layer using a conductive coater (Neoc-ST; Meiwafosis, Tokyo, Japan), and imaged with a SU6600 scanning electron microscope (Hitachi High-Tech, Tokyo, Japan) operated at 5 kV.

### SYTOX Green uptake assay

*E. coli* cultures were adjusted to an OD_600_ of 0.01 and incubated with colistin (10 μg/mL) or Fr-A (fengycin) at 1× or 5× the concentration present in the culture supernatant at 37°C for 4 h. Following treatment, cells were mixed with 0.5 μM SYTOX Green (Thermo Fisher Scientific) and incubated for 5 min at room temperature. Fluorescence kinetics were recorded every 60 s for 30 min at 37°C using a Tecan microplate reader (excitation, 490 nm; emission, 525 nm) in black 96-well plates.

### Statistical analysis

Statistical analyses were performed using GraphPad Prism software (GraphPad Software, San Diego, CA). Data are presented as mean ± standard deviation (SD). Means were compared using one-way ANOVA followed by appropriate post hoc tests (Tukey or Dunnett). Significance was set to *P* < 0.05.

## SUPPLEMENTAL MATERIAL

Supplemental material aem.00276-26-s0001.pdf Fig. S1 to S3; Tables S1 and S2.

ASM does not own the copyrights to Supplemental Material that may be linked to, or accessed through, an article. The authors have granted ASM a non-exclusive, world-wide license to publish the Supplemental Material files. Please contact the corresponding author directly for reuse.
