# Supplementary material for: Role of C17 fengycin B from Bacillus subtilis DB9011 in enterotoxigenic Escherichia coli inhibition
Source: Appl Environ Microbiol. 2026 Jun 4;92(7):e00276-26. doi: 10.1128/aem.00276-26 (PMC13390359; doi:10.1128/aem.00276-26)
Supplement: Supplemental material — Fig. S1 to S3; Tables S1 and S2. [file aem.00276-26-s0001.pdf]

Supplementary data

Role of C17 fengycin B from *Bacillus subtilis* DB9011 in enterotoxigenic *Escherichia coli* inhibition

Indo *et al*.

Supplementary Figures S1–S3

Fig. S1. Antibacterial activity of fractions derived from *B. subtilis* DB9011 culture supernatant against enterotoxigenic *E. coli* (ETEC1263)

Fig. S2. Antibacterial activity of *B. subtilis* DB9011 fractions against *Salmonella* Typhimurium SL1344

Fig. S3. Antibacterial activity of ODS-HPLC 56-57 min and 57-58 min (Fr-A) subfractions against ETEC 1263

Supplementary Tables S1–S2

Table S1. Identification of Fraction A by LC–MS/MS

Table S2. Concentration of C17 fengycin B contained in Fraction-A and culture supernatant

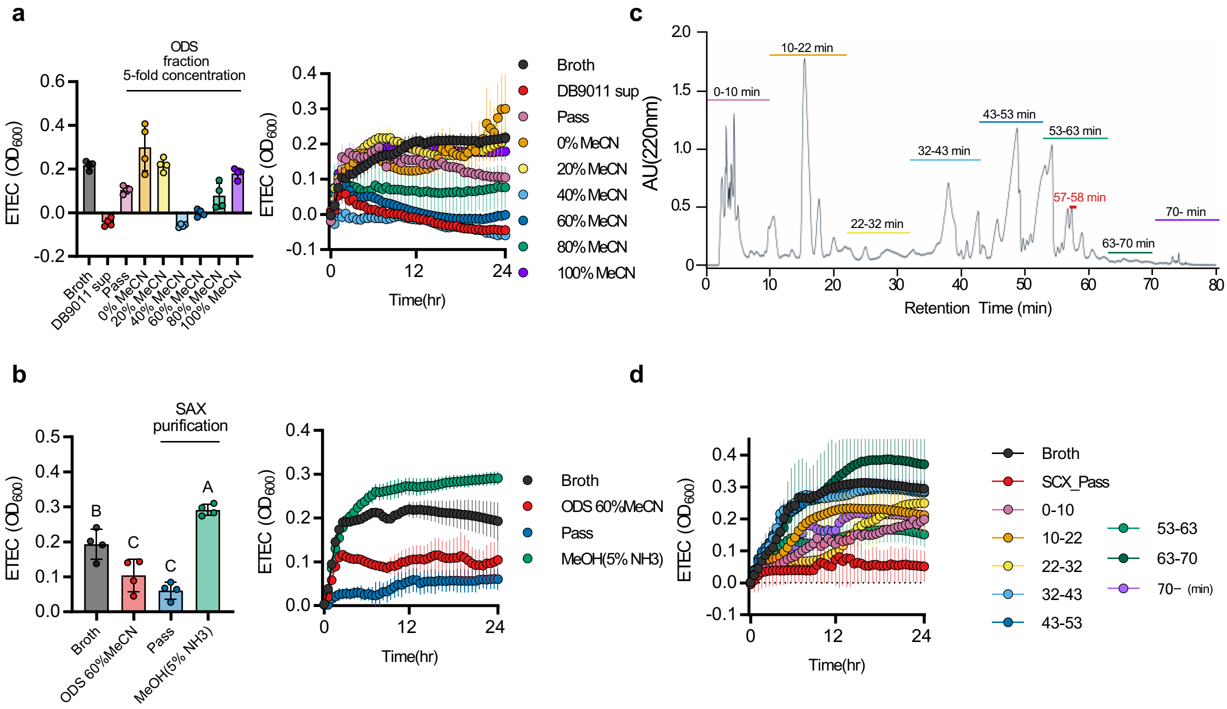

Fig. S1. Antibacterial activity of fractions derived from *B. subtilis* DB9011 culture supernatant against enterotoxigenic *E. coli* (ETEC1263).

(a) Growth inhibition by 5-fold ODS fractions eluted with 0–100% acetonitrile (MeCN). The 40–80% MeCN fraction exhibited strong antibacterial activity.

(b) Activity of strong anion exchange (SAX) fractions. The flow-through fraction retained antibacterial activity, whereas eluates showed reduced effects.

(c) Preparative HPLC chromatography of the strong cation exchange (SCX) flow-through fraction.

(d) Growth curves of ETEC treated with ODS-HPLC fractions collected at 2-min intervals (see Fig. 3d).

Growth was monitored by measuring the OD_{600} over 24 h. Data represent mean ± SD of three independent experiments. Statistical significance (A–C) was determined using one-way ANOVA with Tukey's multiple-comparison test.

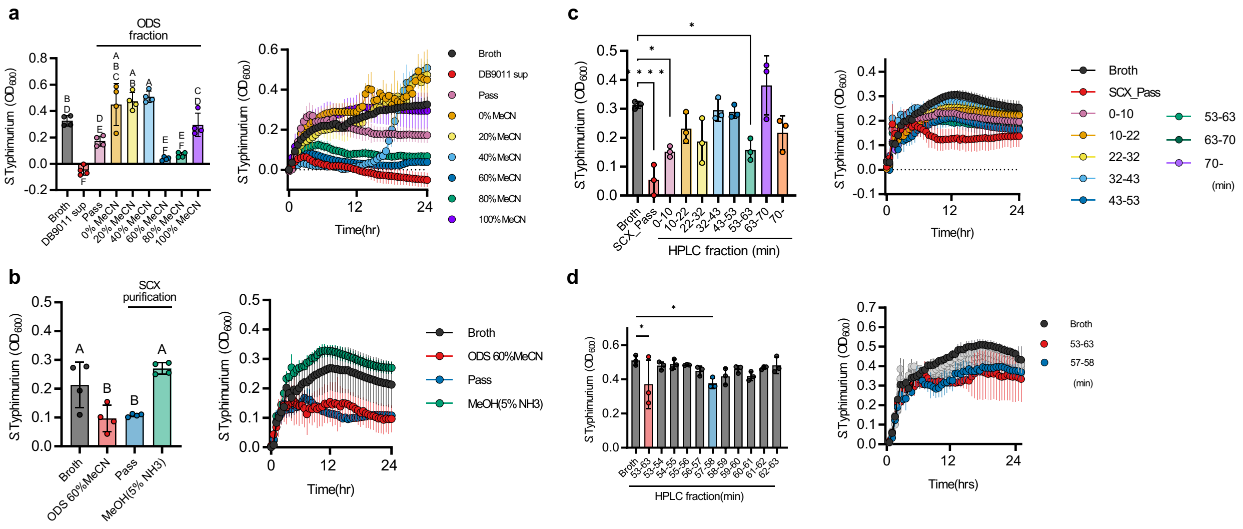

Fig. S2. Antibacterial activity of *B. subtilis* DB9011 fractions against *Salmonella* Typhimurium SL1344.

(a) ODS fractions (0–100% MeCN) were assayed for growth inhibition. The 60% MeCN fraction showed the highest activity.

(b) SCX fractions (flow-through *vs*. 5% ammonia–methanol eluate). The flow-through retained antibacterial activity.

(c) ODS-HPLC fractions eluted at 2-min intervals.

(d) Fine mapping of the 53–63-min window identified the 57–58-min subfraction (Fr-A) as the most active against *S.* Typhimurium.

Bacterial growth was monitored by measuring the OD_{600} over 24 h. Data represent the mean ± SD of three independent experiments. Statistical significance (A–F) was determined using one-way ANOVA with Tukey's multiple-comparison test.

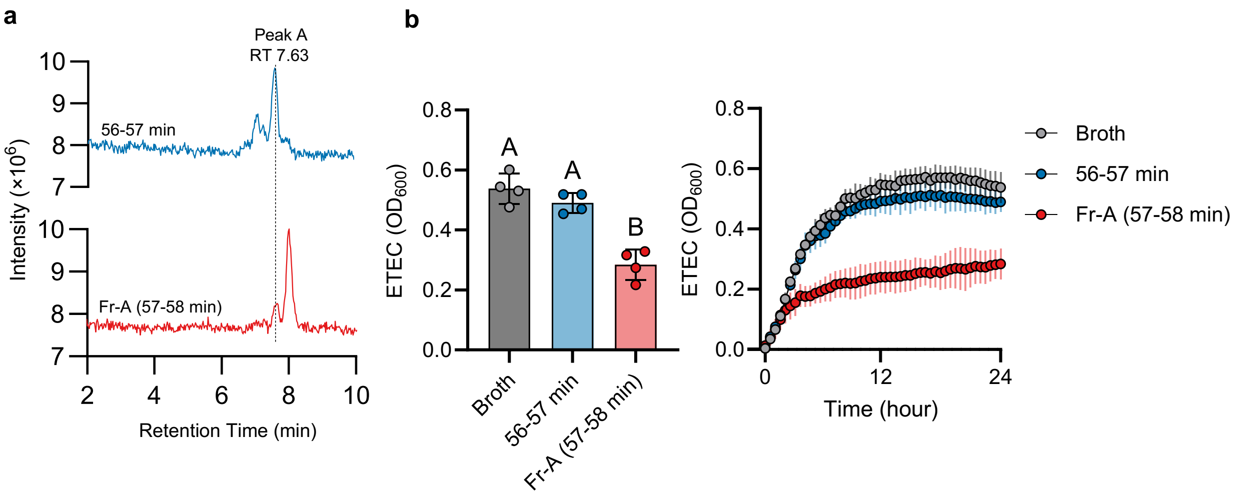

Fig. S3. Antibacterial activity of ODS-HPLC 56-57 min and 57-58 min (Fr-A) subfractions against ETEC 1263

(a) LC chromatograms of the ODS-HPLC 56–57 min subfraction and Fr-A acquired by LC-QTOF/MS in positive ESI mode. Peak A was detected at 7.63 min in both samples.

(b) Antibacterial activity of 56-57 min and Fr-A against ETEC at the same of 5-fold the concentration in the culture supernatant. Right panel shows representative growth curves over 24 h. Data represent the mean ± SD of three independent experiments. Statistical significance (A–B) was determined using one-way ANOVA with Tukey's multiple-comparison test.

Supplementary Table 1
Identification of Fraction A by LC–MS/MS

Precursor ions (*m/z*)	Two fingerprint product ions (*m/z*)	Estimated molecular weight (Da)	Assignment
753.4 ($[M+2H]^{2+}$) and 1505.8($[M+H]^{+}$)	1108 and 994	1505	C-17 fengycin B

Supplementary Table 2
Concentration of C17 fengycin B contained in Fraction-A and culture supernatant

Precursor ions (m/z)	Samples	Dilution ratio	Peak area
m/z 753.4	DB9011 sup	1	190,117
	Fraction-A	100	95,683
m/z 1,503.8	DB9011 sup	1	73,982
	Fraction-A	100	37,734
